# Methoprene-Tolerant (Met) Knockdown in the Adult Female Cockroach, *Diploptera punctata* Completely Inhibits Ovarian Development

**DOI:** 10.1371/journal.pone.0106737

**Published:** 2014-09-08

**Authors:** Elisabeth Marchal, Ekaterina F. Hult, Juan Huang, Zhenguo Pang, Barbara Stay, Stephen S. Tobe

**Affiliations:** 1 Department of Cell and Systems Biology, University of Toronto, Toronto, Canada; 2 Department of Biology, University of Iowa, Iowa City, Iowa, United States of America; University of Cincinnati, United States of America

## Abstract

Independent of the design of the life cycle of any insect, their growth and reproduction are highly choreographed through the action of two versatile hormones: ecdysteroids and juvenile hormones (JH). However, the means by which JH can target tissues and exert its pleiotropic physiological effects is currently still not completely elucidated. Although the identity of the one JH receptor is currently still elusive, recent evidence seems to point to the product of the Methoprene-tolerant gene (Met) as the most likely contender in transducing the action of JH. Studies on the role of this transcription factor have mostly been focused on immature insect stages. In this study we used the viviparous cockroach *Diploptera punctata*, a favorite model in studying JH endocrinology, to examine the role of Met during reproduction. A tissue distribution and developmental profile of transcript levels was determined for Met and its downstream partners during the first gonadotropic cycle of this cockroach. Using RNA interference, our study shows that silencing *Met* results in an arrest of basal oocyte development; vitellogenin is no longer transcribed in the fat body and no longer taken up by the ovary. Patency is not induced in these animals which fail to produce the characteristic profile of JH biosynthesis typical of the first gonadotropic cycle. Moreover, the ultrastructure of the follicle cells showed conspicuous whorls of rough endoplasmic reticulum and a failure to form chorion. Our study describes the role of Met on a cellular and physiological level during insect reproduction, and confirms the role of Met as a key factor in the JH signaling pathway.

## Introduction

Juvenile hormone plays a prominent and diverse role in the regulation of insect development, being implicated in moulting, metamorphosis, reproduction, ageing, polymorphism and caste differentiation in social insects. JH orchestrates insect moulting and metamorphosis together with the ecdysteroids. The hormone is considered to have an anti-metamorphic action i.e. the presence of JH during certain critical periods which results in the continuation of the larval stage. Following the decline in JH titer in the latter half of the final larval stage, an ecdysteroid surge ensues in the absence of JH, and a metamorphic moult takes place. JH rises again in the adult stage of many insects species to regulate female reproductive maturation. Despite the importance of JH, its exact signal transduction pathway remains poorly understood. There is, however, accumulating evidence that Methoprene-tolerant (Met), a basic-helix-loop-helix (bHLH)/Per-Arnt-Sim (PAS) protein, transduces the anti-metamorphic action of JH in both hemimetabolous as well as holometabolous insects [1] and as reviewed by [2], [3]. *Drosophila melanogaster Met* mutants are resistant to toxic doses of JH or its analog methoprene (JHA) [4], [5]. Moreover, both *Drosophila* as well as *Tribolium castaneum* recombinant Met binds JH with nanomolar affinity [6], [7]. Key functional evidence for the anti-metamorphic action of Met was obtained in the red flour beetle, *Tribolium castaneum* in which knockdown of its transcription by RNA interference (RNAi) resulted in a premature larval-pupal metamorphosis and precocious development of adult structures [8], [9].

Recently, the study of the JH signaling pathway has been a prominent topic in insect research and has resulted in the characterization of several factors downstream of the candidate JH receptor, Met, of which Krüppel homolog 1 (Kr-h1) and the Broad-complex (Br-C) are the most studied. Kr-h1, the expression of which is highly dependent on JH, encodes a C_2_,H_2_ zinc-finger type transcription factor [1]. A combination of RNAi experiments and JHA treatments have shown that *Kr-h1* is an early JH-response gene downstream of Met, mediating the anti-metamorphic action of JH in both *Drosophila* and *Tribolium* as well as in Hemimetabola, the cockroach, *Blattella germanica* and the linden bug *Pyrrhocoris apterus* [1], [10]–[13]. The expression of *Br-C*, encoding an ecdysone-regulated member of the Bric-à-brac-Tramtrack Broad (BTB)/Pox virus and Zinc finger (POZ) family of transcription factors, is regulated by JH and can be induced by exogenous methoprene [14]. Br-C was demonstrated to act downstream of the antimetamorphic signaling of JH, being critical for embryonic and nymphal development in Hemimetabola [15]–[18] and specifying pupal features in Holometabola [14], [19], [20].

The elucidation of the JH signaling pathway has mainly been focused on the anti-metamorphic action of this pleiotropic hormone and there are very few reports on the role of Met, Kr-h1 and Br-C during reproduction of insects. These studies have suggested that the role of Met is conserved in different JH-controlled processes in hemimetabolous as well as holometabolous insects, ranging from the antimetamorphic action of JH to oogenesis, oviposition and regulation of Vg mRNA levels, to reduced fecundity, regulation of mating, sex pheromone production and regulation of lipid metabolism [1], [5], [9], [10], [12], [13], [21]–[27]. This suggests that the pleiotropic functions of JH are reliant on a single receptor, but the differing actions may be controlled by partnering or targeting distinct components.

The anti-metamorphic role of JH appears to have been widely conserved in insects with diverse developmental strategies, whereas the role of JH in reproduction appears to have been subject to some modifications. Contradictory data on the exact role of both JH as well as ecdysteroids have been found in insects belonging to different orders. Girardie & Girardie [28] have suggested five patterns to describe the action of different hormones in female reproduction: 1) hormone-independent development; 2) ecdysteroid-dependent; 3) JH and ecdysteroid-dependent; 4) JH and ecdysteroid-dependent with a predominant action of JH and 5) only JH-dependent. In contrast to highly evolved insects, in which endocrine regulation of oocyte development requires both JH and ecdysteroids with Vg synthesis taking place in both fat body and ovary, cockroaches, an order of basal insects, demonstrate a clear correlation between cycles of JH biosynthetic activity by the corpora allata (CA), and oocyte growth and vitellogenesis. Allatectomy and CA implantation experiments have shown that oocyte maturation, fat body vitellogenin (Vg) production and the uptake of Vg by the ovaries are all JH-dependent processes in these animals [29]–[32].

In this study, we examined whether the same key players in the JH pathway responsible for the action of JH are also important in transducing the JH signal in reproduction of the viviparous cockroach, *D. punctata*. Previous work on this animal focused mainly on the physiology of JH, and therefore much is known on the order of reproductive events during the first gonadotropic cycle (as reviewed by [33]). Immediately following the final moult, females mate; this stimulates oocyte growth and JH biosynthesis in the CA. Between day 2 and 3, vitellogenesis commences as the levels of vitellogenin, the precursor of vitellin, rise in the haemolymph and the fat body. When oocytes attain a length of about 0.8 mm, they begin to incorporate vitellogenin from the haemolymph [34]. Elevated JH titer between day 2 and day 4 stimulates this synthesis and uptake [35], [36]. As oocyte growth and vitellogenin accumulation continue, rates of JH biosynthesis begin to decline from day 5. When oocytes reach approximately 1.6 mm in length, the accumulation of vitellogenin slows, the spaces between the follicle cells close and the cells start to deposit chorion [37], [38]. By day 7, the rate of JH production has declined to day 1 levels, choriogenesis is complete and ovulation occurs.

The present study uses RNA interference strategies to examine the role of Met and its downstream targets, Kr-h1 and Br-C in the first gonadotropic cycle of *D. punctata*. This molecular study now verifies that these proteins are key factors in the JH signaling pathway and confirms the important role of JH in vitellogenesis and oocyte development in *D. punctata*.

## Materials and Methods

### Animals

The *D. punctata* colony was maintained at 27–28°C and animals were fed lab chow and water *at libitum*. To obtain pools of synchronized animals, newly molted female adult cockroaches were picked from the colony, placed in separate containers and provided with water and lab chow. Mated status was confirmed by the presence of a spermatophore.

### Tissue collection

*D. punctata* adult females were dissected in sterile cockroach ringer solution (150 mM NaCl, 12 mM KCl, 10 mM CaCl_2_.2H_2_O, 3 mM MgCl_2_.6H_2_O, 10 mM HEPES, 40 mM glucose, pH 7.2) using a dissecting microscope. For each animal, basal oocyte lengths were recorded. Selected tissues were dissected and cleaned of unwanted fat body in sterile saline, flash-frozen in liquid nitrogen to prevent RNA degradation and stored at −80°C until further processing.

### Sequencing of *Met*, *Kr-h1* and *Br-C*

Degenerate primer sequences were designed for *Met*, *Kr-h1* and *Br-C* based on conserved amino acid sequences found upon generating a multiple sequence alignment of several Arthropod orthologs. Primers used for degenerate PCR are listed in Table S1. Partial sequences were obtained using these primers in a standard T-gradient PCR using Taq DNA polymerase (Sigma-Aldrich) and a *D. punctata* pooled cDNA sample containing fat body, epidermis and embryo cDNA. After purification, the resulting DNA fragments were subcloned into a pJET 1.2/blunt cloning vector (CloneJet PCR Cloning Kit, Thermo Scientific) and sequenced following the protocol outlined in the ABI PRISM BigDye Terminator Ready Reaction Cycle Sequencing Kit (Applied Biosystems). For *Met*, the partial sequence was completed using 5′-RACE (Rapid Amplification of cDNA Ends) and 3′-RACE strategies, following the protocol outlined in the Roche 5′/3′ RACE Kit. Primers used for RACE are listed in Table S1.

### Quantitative realtime PCR (q-RT-PCR)

Primers for q-RT-PCR were designed using IDT's (Integrated DNA Technologies) PrimeQuest design tool (http://eu.idtdna.com/PrimerQuest/Home/Index). Primer sets were validated by determining relative standard curves for each gene transcript using a five-fold serial dilution of a calibrator cDNA sample. Efficiency and correlation coefficients (R^2^) were determined for each primer pair. Primers used for q-RT-PCR are listed in Table S2. Reactions were performed in triplicate on a CFX384 Touch Real-Time PCR Detection System (Bio-Rad) as described previously [39]. A dissociation protocol was included to confirm absence of primer dimer formation.

#### Sample preparation

Several tissues were collected from adult females for use in tissue distribution and developmental profiling using q-RT-PCR. Three biologically independent pools of 10 animals each were collected. Pooled samples were homogenised with RNase-free pestles and total RNA was extracted using the RNeasy Lipid Tissue Kit (Qiagen) according to the manufacturer's instructions. An additional DNase treatment (RNase-free DNase set, Qiagen) was performed to eliminate potential genomic DNA contamination. Because of the small size of the CA, RNA from this tissue was extracted using the RNAqueous-Micro Kit (Ambion), followed by the recommended DNase step. Purity and concentration of the resulting RNA samples were measured using a Nanodrop spectrophotometer (Thermo Scientific.). Only intact RNA was used in subsequent PCR reactions. An equal amount of RNA was transcribed in subsequent cDNA synthesis using Superscript III and random hexamers in a final volume of 20 µl following the manufacturer's protocol (Invitrogen Life Technologies). All samples were reverse transcribed together in a single run. The resulting cDNA samples were diluted 10-fold with PCR grade water. A calibrator sample was prepared by pooling 5 µl of each cDNA sample. In the same run, negative control reactions were set up without reverse transcriptase enzyme to test for genomic DNA contamination. To measure the effect of *Met* RNAi on transcript level, fat body was dissected from 6–8 individual animals (n = 6–8). RNA extraction and cDNA sample preparation were performed as described above.

#### Selection of reference genes

Prior to target gene profiling, previously described housekeeping genes [39] were tested for their stability in the designed tissue distribution, temporal profiling and RNAi experiments. The optimal housekeeping genes were selected using geNorm and Normfinder software as described previously [39].

#### Determination of target gene expression

All q-RT-PCR reactions were performed in triplicate on a CFX384 Touch Real-Time PCR Detection System (Bio-Rad) in a 10 µl volume containing 5 µl IQ SYBR Green Supermix (Bio-Rad), 1 µl forward and reverse primer (5 µM), 2 µl of MQ-water and 1 µl of cDNA. A two-step thermal cycling profile was used: 95°C for 3 min, followed by 40 cycles of 95°C for 10 s and 59°C for 30 s. Target specificity was confirmed by running a few representative PCR products on an agarose gel containing GelRed (Biotium). After electrophoresis, only a single band could be visualized using UV, which was further cloned and sequenced (CloneJET PCR Cloning Kit, Fermentas). For each tested cDNA sample, the normalisation factor for the reference genes relative to the calibrator samples was calculated and used to determine the normalised expression levels of the target genes relative to the calibrator, as described by Vandesompele *et al*., 2002 [40].

### RNA interference and methoprene treatment

dsRNA constructs for *Met* and the negative control were prepared using the MEGAscript RNAi Kit (Ambion). The negative control construct (-pJET) was designed in a non-coding region of the pJET 1.2/blunt cloning vector (CloneJet PCR Cloning Kit, Thermo Scientific). T7 promoter sequences were added to the constructs using a single PCR with T7 promoters appended to both PCR primers (see Table S3). These fragments were subcloned and sequenced as described above. Sequences were analysed for presence of the T7 promoter sites and PCR amplicons were used in an overnight, high yield transcription reaction resulting in annealed dsRNA transcripts. Remaining ssRNA and DNA was removed in a nuclease digestion reaction. The RNA was further purified by phase-solid phase adsorption purification, according to the manufacturer's instructions (Ambion). Purity and concentration of the dsRNA was checked with a nanodrop instrument (Thermo Fisher Scientific Inc.). Five-fold diluted dsRNA was run on a 1.2% agarose gel to examine the integrity of the constructs and efficiency of duplex formation.

Adult females were injected with 2 µg of either *Met* dsRNA or -pJET dsRNA diluted in 6 µl of cockroach saline immediately after their final moult (day 0). This treatment was repeated every other day until tissue was assayed. Fat body, CA and ovary were dissected on days 2, 4 and 6 after adult moult. Fat body was immediately stored in liquid nitrogen for RNA extraction. CA were dissected and cleaned in TC199 medium (GIBCO; 1.3 mM Ca2+, 2% Ficoll, methionine-free) for use in the radiochemical assay (RCA). Oocyte length was measured and ovary was collected in 50 µl of Vg extraction buffer (PBS; 0.02 M phosphate, 0.2 M NaCl, pH 7.0).

In a separate RNAi experiment, adult females were injected with *Met* dsRNA on day 0 as described above. On day 2 these animals were given a booster injection of *Met* dsRNA and topically treated with 100 µg of methoprene dissolved in 5 µl of acetone. Control animals were injected with –pJET dsRNA and topically treated with 5 µl acetone.

### Protein & Immunoassays

Single dissected ovaries were homogenised and extracted twice in 50 µl PBS according to Mundall *et al*. [34]. Total ovary protein content was determined using a Bradford [41] assay performed according to the manufacturer's (Sigma-Aldrich) 96-well plate protocol.

Vitellin content was measured using an indirect enzyme-linked immunosorbent assay (ELISA). Vitellin standards were prepared as described by Stoltzman and Stay [42]. Plates were coated overnight at 4°C with unknowns (40 ng/100 µl) and standards (1.56–100 ng/100 µl) in PBS, washed 3 times with PBS-0.05% Tween (PBST) and blocked with 1% BSA in PBS for 30 min. After 3 washes with PBST, plates were incubated for 1 hour at RT with 100 µl/well of a rabbit polyclonal antibody (1∶2000 in 1% BSA) against *D. punctata* mature egg homogenate [42]. Plates were washed 4 times with PBST, incubated for 1 hour at RT with 100 µl/well goat anti-rabbit IgG HRP-linked antibody (1∶3000 in 1% BSA, Cell Signaling), washed 6 times with PBST, and then treated with TMB solution (BioShop) according to the manufacturer. Absorbance was measured at 650 nm with a Molecular Devices SpectraMax Plus 384 microplate reader.

### Microscopy

To examine patency, freshly dissected ovarioles were stained with 1% Evans Blue and processed as described in Pascual *et al*. [43]. To view overall structure, a subset of oocytes were fixed for 3d in 3% glutaraldehyde in 0.1 M phosphate buffer, post fixed for 1 hour with 1% OsO_4_ in 0.1 M phosphate buffer, dehydrated in an ascending ethanol series, and embedded in Spurr's resin. Sections (1 µm) were cut with a Leica EM UC6 ultramicrotome, then mounted and stained with methylene blue and toluidine blue. All light microscopy was performed using a Leica DMI3000 inverted microscope.

For fluorescence imaging, ovarioles were fixed and stained with phalloidin–TRITC (cytoskeleton; Sigma) and DAPI (nuclei; Sigma) as per Cruz *et al*. [44], then examined with a Leica TCS SP8 confocal microscope.

Transverse 100 nm ultrathin sections were taken near the midpoint between the anterior and posterior ends of the basal oocytes embedded in Spurr's resin above. Ultrathin sections were mounted and stained according to [45] and follicle cell ultrastructure was then imaged using a Hitachi HT-7700 transmission electron microscope and AMT Image Capture Engine Software. All images were post processed in Adobe Photoshop.

### Radiochemical assay (RCA)

Rates of JH release were measured using the *in vitro* radiochemical assay (RCA) originally described by Tobe & Pratt [46] and Pratt & Tobe [47]. Day 0 to day 7 female cockroaches were immobilized on ice and their oocytes measured. CA were dissected and transferred to TC199 medium in a sterile environment. A short-term *in vitro* assay for JH release in TC199 medium (GIBCO) [2% Ficoll, 1.3 mM CaCl_2_·2H_2_O and 3 µCi/ml l-[methyl-^14^C]methionine (55 mCi/mmol; American Radiolabeled Chemicals, Inc.) followed by rapid partition was conducted. A single corpus allatum was measured and measurements are means of 8 biologically independent RCA results (n = 8) according to Feyereisen and Tobe [48] and Tobe and Clarke [49].

## Results

### Genes involved in JH signaling are conserved in *D. punctata*

Partial cDNAs encoding the JH receptor Met and its downstream targets Kr-h1 and Br-C were isolated from *D. punctata*. This was accomplished by a RT-PCR approach using degenerate primers based on conserved motifs. For *Met*, 5′-RACE and 3′-RACE experiments were performed to complete the sequence. Blastx National Center for Biotechnology Information (NCBI) database searches revealed that the *Diploptera* orthologs showed substantial similarity to the previously described Met, Kr-h1 and Br-C sequences and were therefore named *DippuMet*, *DippuKr-h1* and *DippuBr-C*. To simplify, these will be referred to as *Met*, *Kr-h1* and *Br-C*. Translation of *Met* cDNA revealed a 950 amino acid protein sequence. Figure S1A shows a multiple sequence alignment of Met with orthologs from different insect species. The main functional domains appear to be well conserved: the basic helix-loop-helix (bHLH) region and two PAS domains: PAS-A and the C-terminal PAS-B, which was recently shown to be sufficient for JH binding [6]. The partial translated amino acid sequence of Kr-h1 contains seven of the eight classical and highly conserved C_2_H_2_ Zn-finger domains and the LP(L/P)RKR motif located at the C-terminus [50] (Figure S1B). The partial amino acid sequence of Br-C contains the BTB domain. *Met* and partial *Kr-h1* and *Br-C* sequences were deposited in the NCBI GenBank and have the following accession numbers respectively: KJ564130, KJ564131 and KJ564132.

### The tissue specificity and temporal expression profile of *Met*, *Kr-h1*, *Br-C* and *Vg* were examined by q-RT-PCR

**Figure 1:**
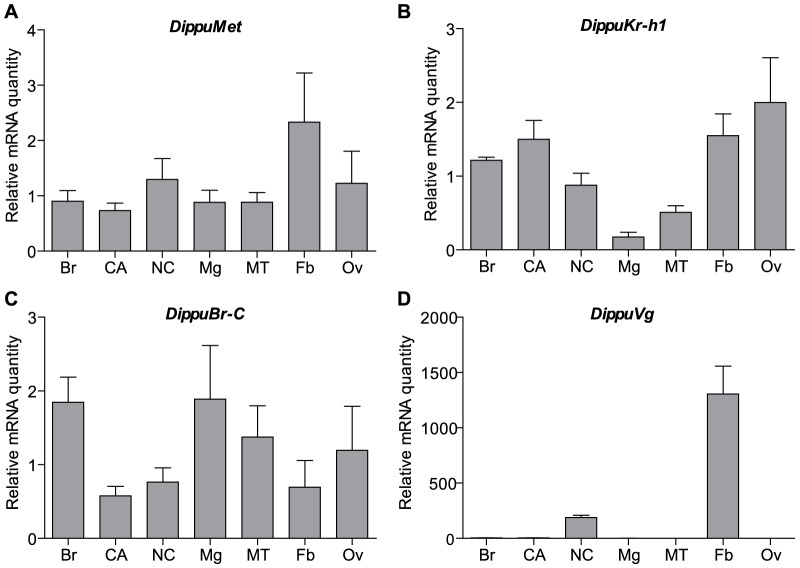
*DippuMet*, *DippuKr-h1*, and *DippuBr-C* are broadly expressed across tissues of *D. punctata*. Relative mRNA quantity of *DippuMet* (A), *DippuKr-h1* (B), *DippuBr-C* (C), and *DippuVg* (D) was measured in the tissues of mated female animals four days after imaginal moult. Grey bars represent the mean over three independent pools of ten animals and technical replicates, normalized to *Tub* and *EF1α*. Vertical error bars indicate SEM. Brain (Br), corpora allata (CA), nerve cord (NC), midgut (Mg), Malpighian tubules (MT), fat body (Fb), and ovary (Ov).

**Figure 2:**
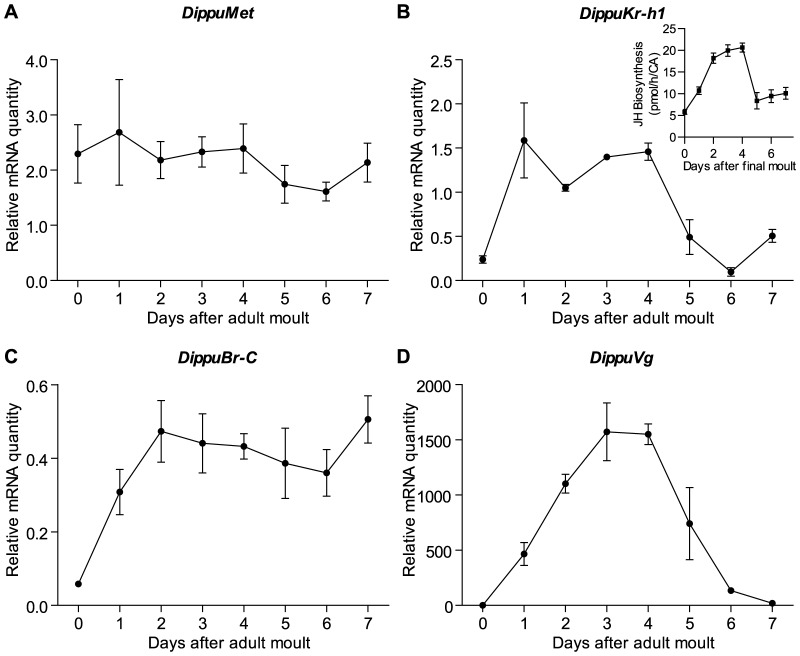
*DippuMet* mRNA levels are stable in the fat body, whereas *DippuKr-h1*, *DippuBr-C* and *DippuVg* fluctuate. Measurements were taken from the fat body each day during the first gonadotropic cycle (day 0 to day 7 after imaginal moult), for target genes *DippuMet* (A), *DippuKr-h1* (B), *DippuBr-C* (C), and *DippuVg* (D). Data points represent the mean over three independent pools of ten animals and technical replicates, normalized to *Tub* and *EF1α*. Inset shows JH biosynthesis per individual corpus allatum for each day during the time period target gene transcript levels were measured in control animals (n = 12). Vertical error bars indicate SEM.

The tissue distribution of *Met*, *Kr-h1* and *Br-C* on day 4 following the adult moult is very broad, with all genes expressed in both nervous tissue as well as peripheral tissues (Figure 1). Expression of *Met* was highest in the cockroach fat body (Figure 1A). For *Kr-h1* high relative mRNA levels were found in the CA, ovary and fat body (Figure 1B). As expected, the expression of *Vg* is limited to the fat body (Figure 1D). The relatively high amount of *Vg* mRNA in the nerve cord is believed to be caused by the remaining presence of difficult-to-remove fat body from this fragile tissue. Since this study focused on the downstream effects of JH on insect reproduction and *Met* and *Kr-h1* mRNA quantities were found to be relatively high in the fat body, the expression of *Met*, *Kr-h1*, *Br*-C and *Vg* was measured every day throughout the first gonadotropic cycle of the female cockroach in this tissue (Figure 2). The developmental profile of *Met* showed the continuous presence of its transcript during oocyte maturation from day 0 to day 7 (Figure 2A). The temporal profile of *Kr-h1*, on the other hand, shows a rapid increase on day 1 and a significant decline on day 5, which correlates with changes in JH biosynthesis in the CA (Figure 2 inset). Similarly, expression of *Br-C* rises rapidly commencing on day 1 and remains high until oviposition. As expected, the expression of *Vg* shows dynamic changes throughout oocyte maturation (Figure 2D), rising on day 1, reaching a maximum on day 4 and dropping dramatically on day 5.

### *Met* is effectively silenced upon dsRNA treatment and the expression levels of JH signaling genes are affected following silencing of *Met*

**Figure 3:**
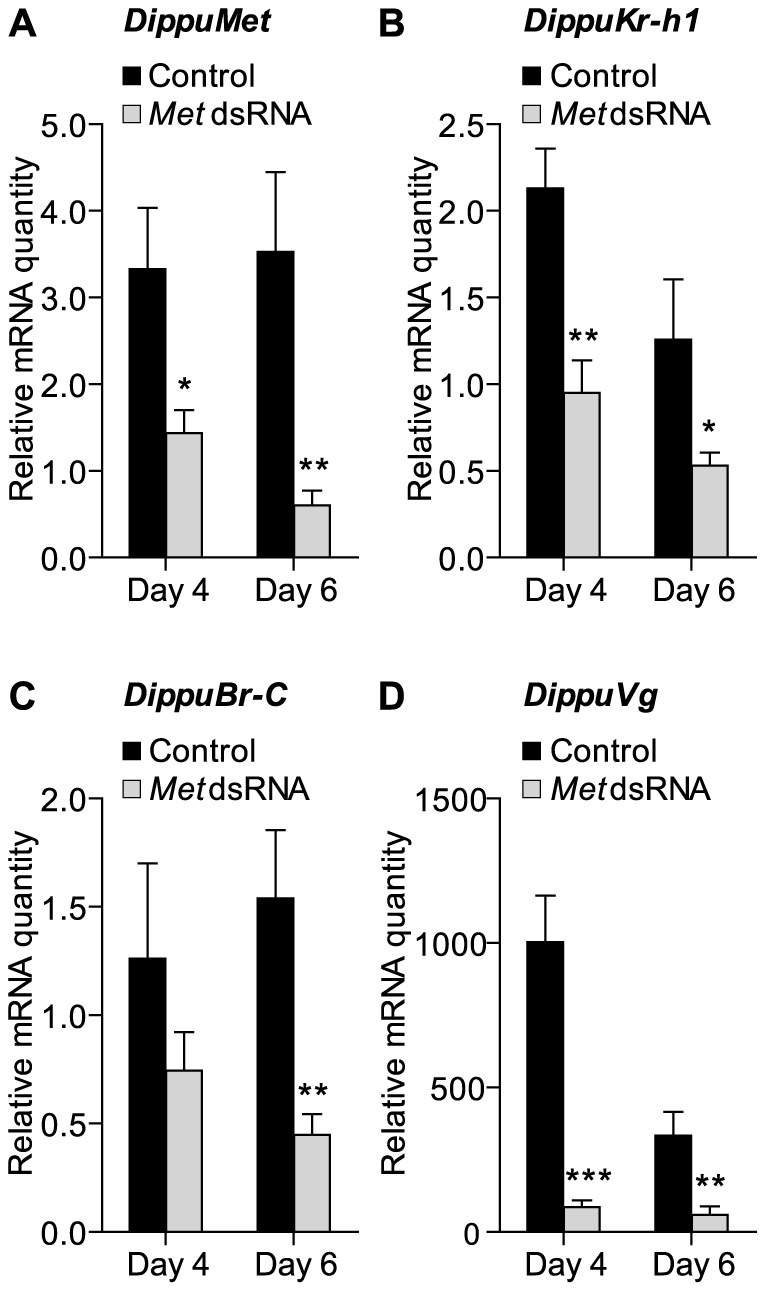
Knockdown of *DippuMet* by RNAi results in significant downregulation of *DippuMet* (A) *DippuKr-h1* (B), *DippuBr-C* (C) and *DippuVg* (D). Measurements were taken from the fat body on days 4 and 6 of the first gonadotropic cycle. Data points represent the mean of 7–8 individual animals, (n = 7–8) and three technical replicates, normalized to *Tub* and *EF1α*. Vertical error bars indicate SEM.

To test whether Met is required in the first gonadotropic cycle of *D. punctata*, RNAi-mediated knockdown of this gene was performed by injecting dsRNA into the haemocoel of day 0 adult females. Two different dsRNA constructs were designed to minimize the probability of off-target effects. Since preliminary experiments yielded the same phenotype, only one construct was chosen to conduct an in depth study. Control animals were injected with a dsRNA construct made in the non-coding region of the pJET 1.2/blunt cloning vector following the same treatment scheme. Two µg of dsRNA were injected every other day starting from day 0. The efficiency of RNAi-mediated knockdown was confirmed using q-RT-PCR (Figure 3). The fat body was assayed on days 4 and 6 of adult female development. As shown in Figure 3A, injection of *Met* dsRNA effectively silenced the transcript level of this gene in treated animals compared to control animals. Transcript levels dropped 57% and 83% on day 4 and day 6, respectively.

#### *Kr-h1* is a potential target downstream of *Met*

Not surprisingly, the expression of target genes downstream of *Met* was also significantly affected upon silencing the JH receptor (Figure 3B–D). Relative mRNA levels for *Kr-h1* were found to be 55% and 58% downregulated on days 4 and 6 respectively. Transcript levels of *Br-C* were not affected on day 4, but on day 6, a 71% knockdown was observed.

#### Silencing *Met* impacts the expression of *Vg*

A very obvious downregulation of expression was observed for *Vg* in the fat body on both days 4 and 6 of adult female maturation, with knockdowns of 91% and 81% in *Met* dsRNA-injected animals compared to control animals (Figure 3D).

### *Met* and *Kr-h1* expression are regulated by JH biosynthesis

Similar effects on transcript levels were found on day 4 of ovarian maturation in animals in which genes encoding rate-limiting enzymes (*HMGR* and *JHAMT*) in the JH biosynthesis pathway were silenced starting from day 0. Rates of JH biosynthesis dropped dramatically, and levels of *Met* and *Kr-h1* were found to be significantly lower in comparison to controls injected with –pJET dsRNA (Figure S2). These findings confirm that the amount of *Met* transcript and that of its downstream target *Kr-h1* is dependent on the rates of JH biosynthesis.

### Blocked basal oocyte development fails to induce the characteristic JH biosynthetic profile during the first gonadotropic cycle

**Figure 4:**
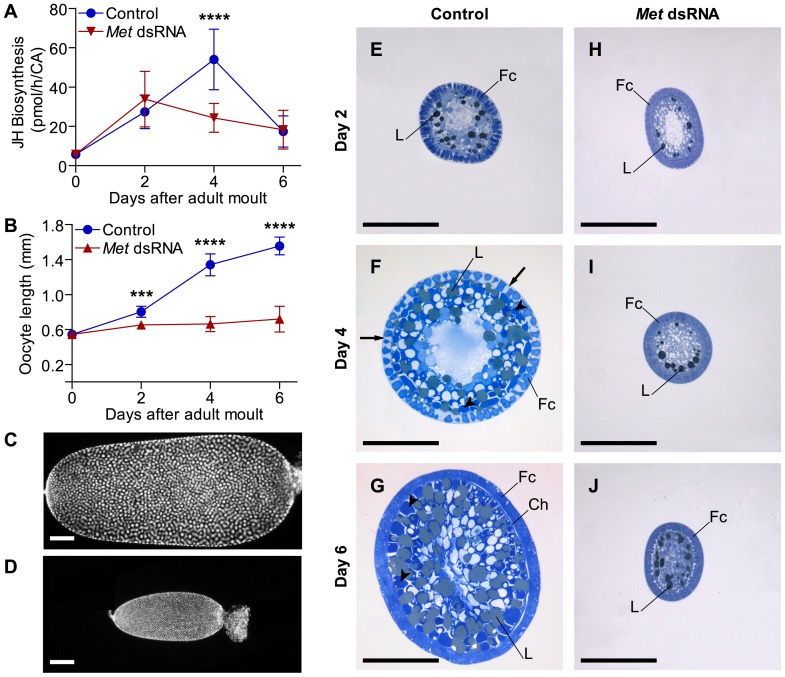
Oocyte growth and JH biosynthesis are inhibited as a consequence of *DippuMet* dsRNA treatment. JH biosynthesis for individual corpus allatum (A) and length of basal oocytes (B) dissected during first gonadotropic cycle of mated female *D. punctata* injected with either control (blue circles) or Met dsRNA (red triangles). Vertical bars indicate SEM, N = 8–20. *C*–*D*, difference in overall size of 6-day-old basal oocyte from control (C) versus *Met* dsRNA (D) treated animals, follicle cell nuclei are stained with DAPI. *E*–*J*, transverse sections of basal oocytes showing follicle cells (Fc), intercellular gaps (arrows), yolk spheres (arrowheads), lipid spheres (L) and chorion (Ch). Scale bars indicate 200 µm.

Changes in JH release from CA in the two treatment groups were analysed using an *in vitro* radiochemical assay (Figure 4A). JH biosynthesis by CA was significantly affected in day 4 animals. *Met* dsRNA treated animals did show the initial rise in JH biosynthesis on day 2, but failed to form the characteristic peak on day 4 observed in control animals (Figure 2 inset and Figure 4A).

### Silencing *Met* by RNAi causes a complete cessation of basal oocyte development

#### Oocytes cease growth in *Met* dsRNA-treated animals

The overall effect of dsRNA treatment on female reproduction was examined by measuring basal oocyte lengths every other day of the gonadotropic cycle. As only the basal oocytes in the *D. punctata* ovary develop during the first cycle, the length of the basal oocyte was used as a measure for oocyte growth and development. Compared to controls, basal oocyte growth was completely arrested on day 0 of the gonadotropic cycle in *Met* dsRNA treated female cockroaches (Figure 4B). The overall size difference of day 6 oocytes in treated (Figure 4D) relative to control (Figure 4C) animals is well illustrated by the staining of the follicle cells of basal oocytes with DAPI. Arrested development became even more obvious when examining transverse sections of the developing ovaries using light microscopy. As seen in Figure 4 E–G, the control oocytes undergo dramatic changes between days 2 and 6. On day 4, large intercellular spaces appear between the follicle cells. This phenomenon, termed ‘patency’, allows the uptake of vitellogenin from the haemolymph into the developing oocytes. As the oocytes attain their full size on day 6, intercellular spaces close and chorion formation takes place. These changes were not observed in the ageing oocytes of *Met* dsRNA treated animals as can be seen in Figure 4H–J. Moreover, ectopic application of methoprene on the *Met* dsRNA-treated animals did not rescue the block of oocyte growth in these animals (Figure S3).

#### Juvenile hormone acts through *Met* to regulate vitellogenesis

**Figure 5:**
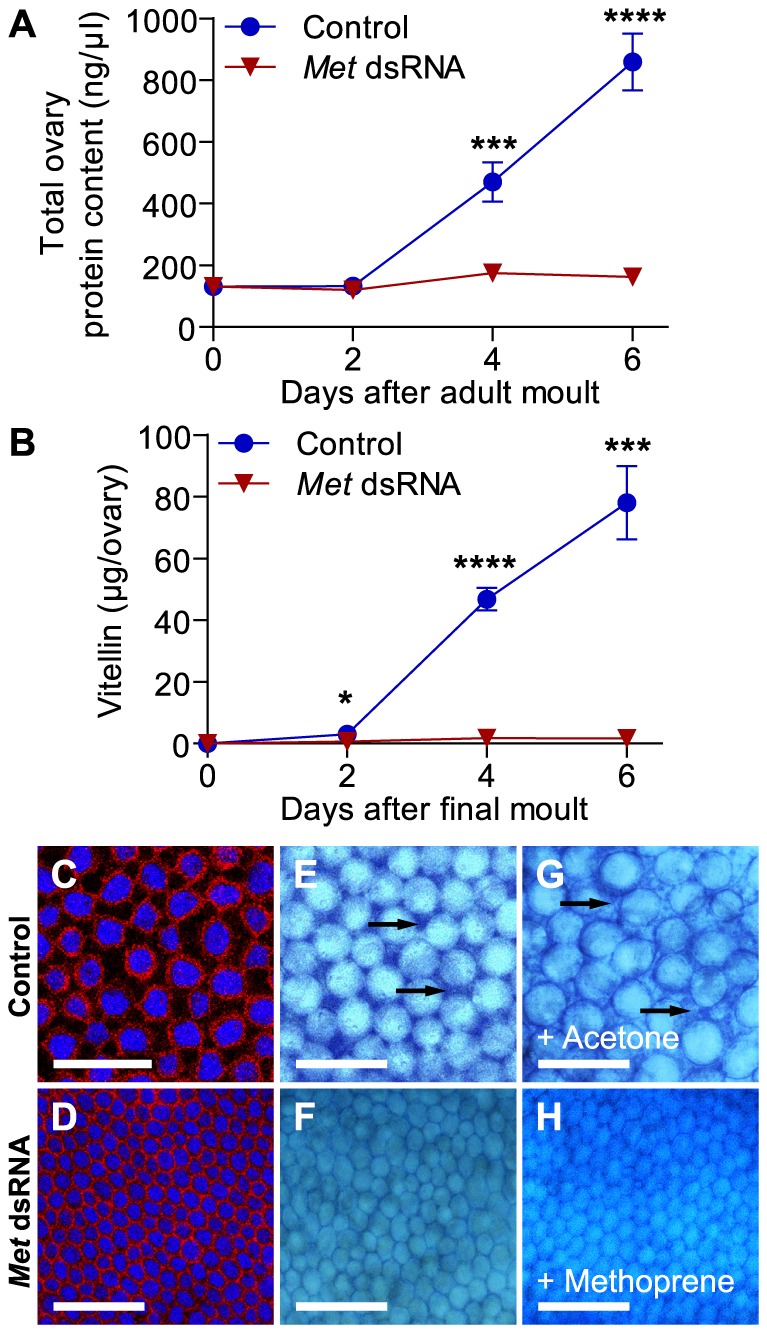
Patency is abolished in *DippuMet* dsRNA-treated animals. Total soluble protein (A) and vitellin (B) extracted from single ovary of control (blue circles) and *DippuMet* dsRNA (red triangles) animals during the first gonadotropic cycle. Vertical bars indicate SEM, N = 5–7. *C*–*H*, morphology of the basal oocyte follicular epithelium from 4-day-old control and *DippuMet* dsRNA ovaries. Follicle cells were fluorescence stained (C–D) for cell nuclei (DAPI, blue), and F-actin (phalloidin-TRITC, red), or stained with Evans blue (E-F) for intercellular spaces (arrows). *G*–*H*, Evans blue staining of control and *DippuMet* dsRNA ovaries dissected from attempted rescue animals with 100 µg topical methoprene. Scale bars indicate 50 µm.

In addition to the significant downregulation of *Vg* mRNA shown above (Figure 3D), dramatic reductions were also observed at the protein level. Total protein and vitellin content were measured every other day from day 0 to day 6 in the developing ovary. Both remained constant in the *Met* dsRNA-treated animals, whereas vitellin content rose dramatically during maturation of the control female ovary (Fig 5A & B). This suggests that knockdown of *Met* resulted in absence of uptake of any vitellogenin that might have been present in the haemolymph.

#### Patency is not induced in *Met* dsRNA treated animals

To further investigate whether patency occurs in control versus *Met* dsRNA treated animals, ovarioles were stained with DAPI and Phalloidin-TRITC to allow for visualization of the morphology of the basal oocyte follicular epithelium using confocal microscopy. On day 4, oocytes from *Met* knockdown animals show smaller tightly packed follicle cells with no interfollicular spaces, in contrast to the control oocytes in which large intercellular spaces occur to facilitate uptake of vitellogenin into the developing oocytes (Figure 5C & D). The lack of patency in *Met* dsRNA-treated animals was further confirmed by examining the degree of Evans Blue penetration into the interfollicular spaces of live, unfixed and freshly dissected oocytes. It was apparent that the basal oocytes dissected from the control animals showed spaces between all the follicle cells on day 4 (Figure 5E). These were not observed between the significantly smaller follicle cells of the *Met* dsRNA treated animals (Figure 5F). Furthermore, application of methoprene did not rescue the failed induction of patency in the *Met* RNAi animals (Figure 5 G–H), thereby strongly suggesting that *Met* is necessary for the action of juvenoids in developing patency in the follicular cell layer of *D. punctata* oocytes.

#### Follicle cell ultrastructure differs and chorion formation does not occur in *Met* RNAi-treated animals

**Figure 6:**
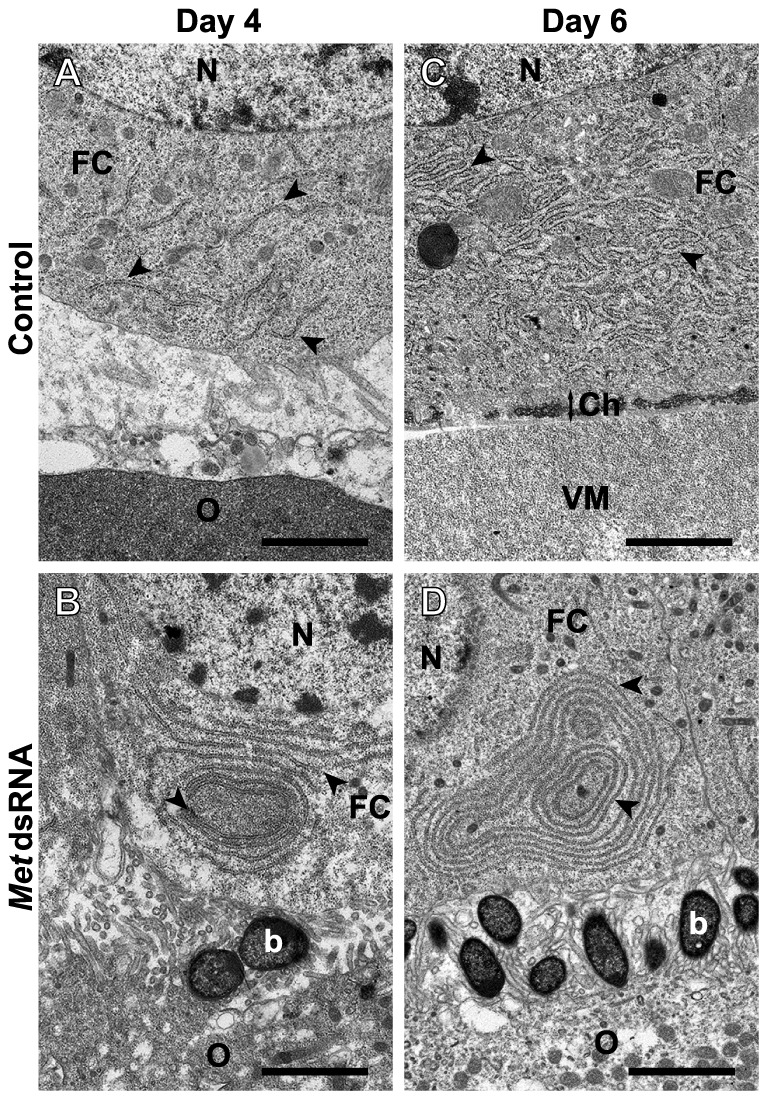
Follicle cell ultrastructure is affected by *DippuMet* dsRNA-treatment. Transmission electron micrographs of the interface between follicle cells (FC) and oocytes (O) from control animals undergoing vitellin uptake (A), early chorion (Ch) formation (C), and *DippuMet* dsRNA-treated animals undergoing autophagic vacuole formation on day 4 (B) and day 6 (D). Plates show the nucleus (N), rough endoplasmic reticulum (arrowheads), bacteroides (b) and vitellin membrane (VM). *A*–*D* are shown at the same magnification; scale bars indicate 2 µm.

To examine the morphology of organelles within the follicle cells, TEM images were studied at several time points during the gonadotropic cycle. By day 4, short strands of rough endoplasmic reticulum (RER) were found in control animals compared to the conspicuous whorls of RER throughout the follicle cells of *Met* dsRNA-treated animals (Figure 6A & B). By day 6, the chorion and vitellin membrane are apparent in controls but do not appear in the treated animals. Short interconnected networks of RER appear in the follicle cells of controls, whereas the encircling membranes of RER remain in the follicle cells of treated animals (Fig. 6C & D). Moreover, bacteroides seem to accumulate in the *Met* dsRNA treated animals. These gram-negative bacteria are destined to enter the oocyte and become incorporated into the mycetocyte cells of the fat body. These bacteria appear more numerous in the *Met* dsRNA treated animals because the basal oocyte remains small, whereas they become less conspicuous as the oocyte increases in size in the control animals.

## Discussion

This study has analyzed the expression and function of the JH receptor, Met, during the first gonadotropic cycle of the viviparous cockroach, *D. punctata*. Based on the results presented, it can be concluded that the *Diploptera* ortholog of the JH receptor Met is a key factor in regulating the vitellogenic action of JH during the first gonadotropic cycle of *D. punctata* and in the transduction of the JH signal.

The distributions of *Met*, *Kr-h1* and *Br-C* in the tissues of *D. punctata* show that these genes are expressed ubiquitously in both nervous and peripheral tissues, as was previously reported for *Kr-h1* in larval *B. germanica* [10]. This is consistent with the role of these genes in transducing the signal of the pleiotropic JH. Moreover, temporal expression profiles of *Met* and *Kr-h1* in the fat body are consistent with previous reports examining the expression of these genes in larval stages. *Met* mRNA is continuously present, with little major fluctuation, whereas the temporal profile of the *Kr-h1* transcript appears to be more dynamic, responding to changes in circulating JH titer [1], [10], [11], [24], [52]. Relative expression of *Kr-h1* closely follows the increase and decrease in the rates of JH biosynthesis by the CA. Alignments of the amino acid sequences of Met, Kr-h1 and Br-C reveal a high degree of conservation in the main functional domains of these proteins, previously reported to play key roles in transducing the antimetamorphic JH signal of other insects [1], [2], [8]–[12], [14], [19], [24], [51].

Our results demonstrate that *Kr-h1* expression is highly dependent on *Met* and JH, whereas *Br-C* expression is not. Silencing *Met* not only resulted in a significant reduction of *Met* mRNA expression levels, but also of its downstream target *Kr-h1*. Similar results were reported in a very recent study focused on the reproductive cycle of *P. apterus* [27]. This was not apparent for *Br-C* for which expression rises after adult moult and stays high throughout the first gonadotropic cycle. Expression of *Br-C* seems to be much less dependent on JH. Knockdown of *Met* only resulted in a downregulation of *Br-C* expression on day 6, when JH production is low. Furthermore, silencing of *HMGR* and *JHAMT*, two genes considered to encode rate-limiting enzymes in the JH biosynthetic pathway, yielded a similar outcome. Both *Met* and *Kr-h1* were significantly downregulated, but not *Br-C.* Our study is consistent with previous reports studying immature stages of the hemimetabolous *P. apterus*, in which knockdown of *Met* showed a strong dependence of *Kr-h1* expression on *Met*, which was not clearly observed for *Br-C* [1]. Further study is needed to identify the exact role of *Br-C* during cockroach reproduction.

Since ovarian maturation is dependent on JH in *D. punctata* females, this animal may be an ideal model for analyzing whether the function of JH in reproduction employs the same or a similar molecular signaling pathway as occurs in metamorphosis. In this animal, JH is known to regulate oocyte growth, vitellogenin production in the fat body and uptake of vitellogenin by the basal oocytes [29]–[32]. Results from the knockdown of *Met* during the first gonadotropic cycle indicate that *Met* is a key player in JH signal transduction during reproduction. Silencing of *Met* resulted in a complete block of basal oocyte development. Treatment with *Met* dsRNA not only resulted in the downregulation of *Vg* mRNA quantities in the fat body, but also suppressed the induction of patency and uptake of vitellogenin into the developing oocytes, and arrested basal oocyte growth. Moreover, knockdown of *Met* also affected the ultrastructure of the follicle cells. In treated animals, neither the chorion nor vitellin membrane was observed and whorls of RER were found throughout follicle cells. Circular formations of ER are linked to the early stages of autophagy, whereas short strands of RER, as seen in controls, are associated with active protein synthesis [53]. The inactive CA of ovariectomized mated females show whorls of smooth ER, similar to the RER structures observed in the *Met* dsRNA-treated follicle cells (50). It is known that stimulation from the developing ovary is required for morphological changes in the mitochondria and smooth ER associated with increased JH biosynthesis in the CA [54]. This suggests that *Met* may be involved in communication, or feedback between the CA and ovary that promotes cellular activity and protein synthesis.

The effect of *Met* dsRNA-treatment on rates of JH biosynthesis in *D. punctata* provides insights into these feedback mechanisms acting between the CA and ovary. A very recent study showed that the transcription of genes directly involved in the JH biosynthetic pathway closely follow CA activity in *D. punctata* (Huang, Marchal, Hult and Tobe, unpublished results, data available upon request). Furthermore, silencing of two of these genes –*HMGR* and *JHAMT* – effectively abolished JH biosynthesis, downregulated *Vg* expression in the fat body and significantly reduced oocyte growth and the induction of patency (Huang, Marchal, Hult and Tobe., unpublished results, data available upon request). This result was expected, as both JH biosynthesis and *Vg* transcription require a functional JH biosynthetic pathway. However, *Met* dsRNA-treatment also had a significant effect on rates of JH biosynthesis. Treated animals failed to produce the characteristic peak of JH on day 4 of the first gonadotropic cycle. Since the RNAi response appears to be systemic in *D. punctata*, *Met* would also be knocked down in the CA of these animals. Therefore, it is possible that the positive feedback mechanism by which JH produced by the CA stimulates its own biosynthesis, likely through its action on the maturing ovary and the subsequent release of a stimulatory factor from the ovary [55].

The normal pattern of JH biosynthesis in the CA during the first gonadotropic cycle of mated females (as seen in Figure 2 inset) is known to require the presence of the ovary [30], [32], [56]. Previous reports have shown that ovariectomy of females soon after the adult molt results in a low level of JH biosynthesis throughout the first gonadotropic cycle [32]. Furthermore, Rankin and Stay [30] have shown that the size of the basal oocytes is a good indicator of the degree to which the developing ovary can induce JH biosynthesis. The ovary acquires this ability at the start of vitellogenesis but loses it post-vitellogenesis. At present, the exact nature of the ovarian factor(s) involved in this modulation of JH production is unknown. There is however a report that a stage-specific peptidergic factor from the ovary can stimulate JH production [55]. This ovarian factor is produced by basal oocytes ranging in length from 0.76 to 1.15 mm (in day 2 and 3 adult females), which is consistent with our results showing a failure to induce the characteristic pattern of JH biosynthesis following the blockage of ovarian development by silencing *Met*. It is currently unknown whether JH itself or the presence of Vg in the haemolymph induces patency. However, oocytes in the *Met* dsRNA treated animals do reach a size at which they are competent to take up Vg from the haemolymph [30]. It can therefore be suggested that the failure to induce patency does not result indirectly from a lack of oocyte development, but is a consequence of a decreased JH titer and blocked JH signal transduction.

While it is clear that *Met* is critical for vitellogenesis in *D. punctata*, there is likely to be cross talk with other pathways. Recent studies have identified that not only JH, but also nutritional signals, mediated by the very conserved insulin-like peptide (ILP)/TOR pathways, act as key regulators in insect vitellogenesis [25], [57]–[59]. A detailed study in *Tribolium* revealed that JH (signaling) acts upstream of insulin signaling. RNAi-induced silencing of JH biosynthesis (*JHAMT*) or signaling (*Met*) in *T. castaneum* resulted in decreased expression of genes encoding ILPs. Moreover, *Vg* expression was severely impaired by reducing phosphorylation of the ILP downstream factor FOXO (fork head transcription factor). Dephosphorylated FOXO inside of the nucleus was shown to bind a DNA response element in the *Vg* promoter, thereby possibly directly regulating its transcription. These effects could be rescued by injection of bovine insulin. Studies in the mosquito, *Aedes aegypti* and the cockroach, *Blattella germanica*, have demonstrated feedback of the ILP/TOR pathways on the JH biosynthetic activity of the CA, thus placing JH downstream of the ILP/TOR pathway [60]–[62]. In starved *B. germanica*, silencing *FOXO* resulted in increased *Vg* mRNA levels in the fat body and a rise in JH biosynthetic activity. These reports underline the intricate interplay between nutritional signals and JH in the reproductive events in the fat body of insects. At this point, further study will be needed to investigate the involvement of the ILP/TOR pathway in the regulation of vitellogenesis in *D. punctata*.

In summary, our data show conclusively that knock down of the expression of *Met* at the start of the first gonadotropic cycle effectively blocks basal oocyte development and maturation, as indicated by smaller basal oocytes, a dramatic reduction in the expression of vitellogenin in the fat body, a failure to induce patency and the lack of uptake of vitellogenin into the developing oocytes. We conclude that *Met* and its downstream partner *Kr-h1* are essential factors in the JH signal transduction pathway during the first gonadotropic cycle of the cockroach, *D. punctata.*

## Supporting Information

Figure S1 **Amino acid sequence alignment of** ***Diploptera*** **proteins with several functionally characterized ortholog insect proteins** (A) DippuMet with Met from the red flour beetle, *T. castaneum* ([8], [9], GenBank accession number NP_001092812.1), the fruit fly *D. melanogaster* ([4], [5], [7], Genbank accession numbers NP_511126.2 and NP_511160.2), the migratory locust, *Locusta migratoria* (GenBank accession number the linden bug, *Pyrrhocoris apterus* ([1], GenBank accession number AEW22976.1) and the blood-sucking bug *Rhodnius prolixus* ([1], GenBank accession number AEW22977.1). The main functional domains are indicated: the basic helix-loop-helix (bHLH) region and two PAS domains (PAS-A and PAS-B). Eight amino acids in the PAS-B domain involved with JHIII binding in *Tribolium* Met are indicated with asterisks [6]. (B) DippuKr-h1 with Kr-h1 proteins from the red flour beetle, *T. castaneum* ([11], GenBank accession number NP_001129235.1), the fruit fly *D. melanogaster* ([12], GenBank accession number CAA06544.2), the linden bug, *P. apterus* ([1], GenBank accession number AEW22979.1), the blood-sucking bug *R. prolixus* ([1], GenBank accession number AEW22980.1) and the German cockroach *B. germanica* ([10], GenBank accession number CCC55948.1). The eight zinc-finger motifs are indicated. (C) DippuBr-C with Br-C proteins from the red flour beetle, *T. castaneum* ([14], [19]. GenBank accession number NP_001104734.1), the fruit fly *D. melanogaster* (as reviewed by [2] GenBank accession number CAA38476.1), the linden bug, *P. apterus* ([1], GenBank accession number AEW22982.1) and the German cockroach *B. germanica* [18].
(PDF)

Figure S2 **Knockdown of** ***DippuHMGR-DippuJHAMT*** **by RNAi results in significant downregulation of** ***DippuMet*** **,** ***DippuKr-h1*** **,** ***DippuVg***. Measurements were taken from the fat body on day 4 of the first gonadotropic cycle. Data points represent the mean 5 individual animals, (n = 5) and three technical replicates, normalized to *Tub* and *EF1α*. Vertical error bars indicate SEM.
(PDF)

Figure S3 **Effect of topical application of methoprene on oocyte growth** in *DippuMet* dsRNA treated animals versus control animals (n = 5). Plus sign (+) indicates whether animals were treated with acetone or 100 µg of methoprene.
(PDF)

Table S1 **Degenerate primer and RACE primer sequences for cloning partial** ***DippuMet*** **,** ***DippuKr-h1*** **and** ***DippuBr-C*** **cDNAs.**
(DOCX)

Table S2 **Oligonucleotide sequences for primers used in q-RT-PCR for reference and target genes. Efficiencies and R^2^ values are indicated.**
(DOCX)

Table S3 **Nucleotide sequences of primers used in making the dsRNA constructs.**
(DOCX)
