# Supplementary material for: Methoprene-Tolerant (Met) Knockdown in the Adult Female Cockroach, Diploptera punctata Completely Inhibits Ovarian Development
Source: PLoS One. 2014 Sep 8;9(9):e106737. doi: 10.1371/journal.pone.0106737 (PMC4157775; doi:10.1371/journal.pone.0106737)
Supplement: Figure S1 — Amino acid sequence alignment of Diploptera proteins with several functionally characterized ortholog insect proteins (A) DippuMet with Met from the red flour beetle, T. castaneum ([8], [9], GenBank accession number NP_001092812.1), the fruit fly D. melanogaster ([4], [5], [7], Genbank accession numbers NP_511126.2 and NP_511160.2), the migratory locust, Locusta migratoria (GenBank accession number the linden bug, Pyrrhocoris apterus ([1], GenBank accession number AEW22976.1) and the blood-sucking bug Rhodnius prolixus ([1], GenBank accession number AEW22977.1). The main functional domains are indicated: the basic helix-loop-helix (bHLH) region and two PAS domains (PAS-A and PAS-B). Eight amino acids in the PAS-B domain involved with JHIII binding in Tribolium Met are indicated with asterisks [6]. (B) DippuKr-h1 with Kr-h1 proteins from the red flour beetle, T. castaneum ([11], GenBank accession number NP_001129235.1), the fruit fly D. melanogaster ([12], GenBank accession number CAA06544.2), the linden bug, P. apterus ([1], GenBank accession number AEW22979.1), the blood-sucking bug R. prolixus ([1], GenBank accession number AEW22980.1) and the German cockroach B. germanica ([10], GenBank accession number CCC55948.1). The eight zinc-finger motifs are indicated. (C) DippuBr-C with Br-C proteins from the red flour beetle, T. castaneum ([14], [19]. GenBank accession number NP_001104734.1), the fruit fly D. melanogaster (as reviewed by [2] GenBank accession number CAA38476.1), the linden bug, P. apterus ([1], GenBank accession number AEW22982.1) and the German cockroach B. germanica [18]. (PDF) [file pone.0106737.s001.pdf]

```
DippuMet    1 --------------MLGWISPPQLPMGALTIDNMNYSRQNRNMMEKHRRDKMNAHISNLA
TricaMet    1 --------MDSGDCETEEDSAPEACVSPEGSLLPSNSREMRNRAEKMRRDKLNSYIGELA
DromeMet    1 MAAPETGNTGSTGSAGSTGSGSGSGSGSGSSSDPANGREARNLAEKQRRDKLNASIQELA
LocmiMet    1 -----------MISWEHYVPPPPPPDVYHELCPVSNNRQMRNKAEKQRRDKLNSLITELA
PyrapMet    1 ------------------------------------------------------------
RhoprMet    1 -----------------------------------------------------QFISELA

DippuMet   47 LLVPTVANSPKKMDKTSILRLTAAFLRLHKFLVTDPGELN------------RIDFPNFL
TricaMet   53 TLVPMVARSAKRMDKTSILRLAATHLRIYQTLLSGKNHPH-------------IQLPKHV
DromeMet   61 TMVPHAAESSRRLDKTAVLRFATHGLRLQYVFGKSASRRRKKTGLKGTGMSASPVGDLPN
LocmiMet   50 HLVPMVAQSPRRMDKTSILRLSATFLRMYRCLPLKPGEMHE----------PFLPFLETL
PyrapMet    1 --VPMVSSSPKKLDKTSILRLSATFLRLSQFSKQSKE----------------IAEVKES
RhoprMet    8 VLVPMVASSAKKLDKTSILRLSASYLRMHHVAKSTKE----------------VTIKKLS

                         bHLH
DippuMet   95 KDCNLSQAFMEVMDGFMIIVSCSGNILFVSHTVETLLGHPQTSLLGQKLHSITCREDHDT
TricaMet  100 DQYLLEQLVCEQLGGFLLILTPNGKIVFVSHTVEHLLGHLQTDLMGQSIFNITSPDDHDR
DromeMet  121 PSLHLTDTLMQLLDCCFLTLTCSGQIVLVSTSVEQLLGHCQSDLYGQNLLQITHPDDQDL
LocmiMet  100 KVSQVLLDFLQGMEGFMLVVTGAGKIVFVTHTVEKLLGHTQNDLLGQSLYNITRPEDHEE
PyrapMet   43 VKPDWCHRVLDDMEGMLLVVSSTGKIVYVTENVEKVLRHHQNDLLGQSLYDSTHPEDHII
RhoprMet   52 VPINWCHCVLDVMGGILLIVTSSGKIVFISSHVEGLLGYQTNDLLGQSLFTITSPEDRDK

                                                 PAS-A
DippuMet  155 LNKNLNPDPDPSAGDQENSGQLSVGEDSSSSGDTSNSSSPSVPSPQQQQ-----------
TricaMet  160 LRMYINTESVLDG-----------------------------------------------
DromeMet  181 LRQQLIPRDIETLFYQHQHHQQQGHNPQQHSTSTSASTSGSDLEEEEMETEEHRLGRQQG
LocmiMet  160 LRRNIETAPSDGQGRAAAGCSPALTSDESSCEDGAAGGSTPGSSPSASRPGTSASTASVG
PyrapMet  103 LRHNLKPSEEGG------------------------------------------------
RhoprMet  112 LKMNLKPDFDPD------------------------------------------------

DippuMet  204 -------------QQLQPSTSQQQEAPSEPLRQRRSFYLRLSQRTVS-------------
TricaMet  173 -------------------------------DWKKCFNIRLKRAGPR-------------
DromeMet  241 EADDDEDHPYNRRTPSPRRMAHLATIDDRLRMDRRCFTVRLARASTR-------------
LocmiMet  220 SS-----------SDGGRTGPGGPFGSAGAEHLRRSFYVHLAQRAGGRAASSASGTGGGS
PyrapMet  115 --------------DLSRFPE------------RRSFMMRVKLRAAS-------------
RhoprMet  124 --------------LPSTSSEEDILNLEGMKRTRRSFFLKLQHRAIS-------------

DippuMet  238 -----------------RSEITQYELMHVLGHLRVPQRPSQGPSTQSRTRSRQRDGSSTG
TricaMet  189 ------------------TESAVYEPVRIMGVHRPGFDNDCNKNTS-------TSKEIAL
DromeMet  288 -----------------AEATRHYERVKIDGCFRRSDSSLTGGAAANYPIVSQLIRRSRN
LocmiMet  269 SGGGSGGGGGSVGVAGQGAEQTTYELVHFLGYLQVLRSHDPPPATHRPRRHRDTVPSSNS
PyrapMet  136 ----------------RSDQQPQYEVIQLQGHLRSPRNNQTQQRSKSHAFERYLKTHLSV
RhoprMet  157 ----------------KSDQ-PQYEDVHIEGHLRIPPGSASNKKQK--------GEHLTN

DippuMet  281 SSVDS------------------------------DTVLIAVVRTVRESS----------
TricaMet  224 NN----------------------------------DVLLFFVKVFRPEP----------
DromeMet  331 NNMLAAAAAVAAEAATVPPQHDAIAQAALHGISGNDIVLVAMARVLREERPPEETEGTVG
LocmiMet  329 DN-----------------------------------VLVAVVQLFREKR----------
PyrapMet  180 TNGSSFNDTQGEGGS-------------------NEIFLVALVRLYSLKH----------
RhoprMet  192 DN----------------------------------VVLVAVMKPCREKR----------

                           * *       *                 *                *
DippuMet  301 VAERSLLEPSKDEYVTRHLIDGRIIYSDHRISVVAGYLAEEVAGESAFKFMHRDDVRFTI
TricaMet  240 LCER-LFEASREEYVTRHLIDGRIIGCDQRISFIAGYMTEEVSGLSAFKFMHREDVRWVM
DromeMet  391 LTIYRQPEPYQLEYHTRHLIDGSIIDCDQRIGLVAGYMKDEVRNLSPFCFMHLDDVRWVI
LocmiMet  344 VMELSLLEATLDEYQTRHLKDGSIVSSDHRISVVAGYLTDEVHMQNAFTFMHHDDMAYAL
PyrapMet  211 VMDNSVLHAYKGEWITRHMLDGTIVNADHRISILSGYLAEEVSGDSAFKYVHEDDVRWVM
RhoprMet  208 ITAHSILEATKEEWISRHLIDGTIVYSDHRISVVSGYLAHEVSGSPAFLYMHSDDVRWVM
```

```
                                  *           *                 *
DippuMet  361 VALRQMYDRGKDSYGSSCYRLLCKTGQYIYMRTHGYLEYDKDTQQIVSFICINTLVPEDE
TricaMet  299 IALRQMYDRGE-SKGSSCYRLLSRNGQFIYLRTFGFLEIDDQGT-VESFVCVNTLVSEQE
DromeMet  451 VALRQMYDCNS-DYGESCYRLLSRNGRFIYLHTKGFLEVDRGSNKVHSFLCVNTLLDEEA
LocmiMet  404 IALQQMFDKNE-PFGSSTYRLATKNGQYIYMRTRGYLEFAPGSKEVETFLCINTLLSKEE
PyrapMet  271 VALRQMYSSEK-KSGWSCYRLRSKNGDVIYLRSEGYIIYDDDDK--TSFICINTLVSSDD
RhoprMet  268 IVLRQMYYKGE-SYGSSCYRLLSKNGEFIYIRTHGYLELSGEDNSFQSFICINSLVSPEE

                                          PAS-B
DippuMet  421 GETLVQEMKTRYSATVMSSRQQAALLG---------PVPGPSTSTGSSSVEADPLDAAIS
TricaMet  357 GLQLINEMKKRYSALINSQSCP-------------------ITSSGSTDSSS--------
DromeMet  510 GRQKVQEMKEKFSTIIKAEMPTQ------------------SSSPDLPASQAP-------
LocmiMet  463 GEEGLREMKQRFSAYVSALRAAGADLSLTGIAAAIAAAPPPFRSSAVTASERPTLAPPPP
PyrapMet  328 GEKMIREMKNSFCNFFQEKAMY------------------LGLEPGFSGTIKPKLTPSPQ
RhoprMet  327 GEKLIAQMKAQFAPIVMQ-----------------------SNEPGTSALADTRAIMDSS

DippuMet  472 QLITNIPEVRDDGKSEPSSLPDTQYVKAVRYSKVLPSVTEQAAKVGIKNVIPTTRGGPTS
TricaMet  390 --------------------------------QSVEDPQQVEAAIVHLIANLPS---PGS
DromeMet  545 --------------------------------------QQLERIVLYLIENLQKSVDSAE
LocmiMet  523 PPPPPLQPPPPPPPPPPPAPPTAANSLSSSEIISNTTVCEVSPPFTAMVTESTPSSEQGA
PyrapMet  370 ESAINLQR------------------------LKCNDKIALTTAIDMLLCKFHRNMPRFS
RhoprMet  364 -PTPNISP------------------------LKVDDPNELRTAIEQLLTEVPSSEVQLS

DippuMet  532 PTGPLTVAIPEGPQKSQNEANWIHVKRESVITSLVPAVKVEVVRRQETVLVSAQSDSSSD
TricaMet  415 DQRSTPSPRVYGNVNENQDCSTPTENSPTKPYYKLKTANNK----RPPSTELGTNIYTSS
DromeMet  567 TVGGQGMESLMDDGYSSPANTLTLEELAPSPTPALALVPPAPSSVKSSISKSVSVVNVTA
LocmiMet  583 DVFGSVSPSEAAASKYRNHTPTTASRSPTADPYLPAARPRAVSEGEDTTAISLQNFVNLF
PyrapMet  406 ELR--INLHCSNYVKSS------SEDEEQSNEKQLPSSQGR--YSRPSVITSITGSTERQ
RhoprMet  399 PESPVPNQQFAKIAKESKNMPPVTIQSSRIGVMSVPCLKKGPIYNRPSVITPLPRSKDKS

DippuMet  592 VSRLRGNNAVLKRTSSVADCEVQTGSKRQHVSYARRRERRSTEPTSQHQPKSPTLEEPQR
TricaMet  471 KRQRTSPQLSPMSSLPPYPNRTQITEVT--------------------------------
DromeMet  627 ARKFQQEHQKQRERDREQLKERTNSTQG-----------------------------VIR
LocmiMet  643 KVPEEEEHSVDVQRSPLVNNTGVISDLE--------------EKPRITNSLSPSSPPSRH
PyrapMet  456 KRSQENSEALEVKRFRAESRSVIVSDIR--------------TVIRRKEP---------D
RhoprMet  459 KRGVNEEKVSVLKRIRPEETSVIRTVAR--------------EDAGLQPPSCQEQVPPSH

DippuMet  652 TPEEQLRLIMSRNSSVTSPSSNSPLHCIGRG-----------------------------
TricaMet  499 --QQTEPSIYQHDQLLRNKV----------------------------------------
DromeMet  658 QLSSCLSEAETASCILSPASSLSASEAPDTP-----------------------------
LocmiMet  689 PYAAAQRIRAASESELQAANSGSTFIQSDPADRGCSWQFQRHGDVSHSGTTDRRPPERPT
PyrapMet  493 AYDLDTSFMFNADMLMSSASSSCLSNISD-------------------------------
RhoprMet  505 QSHYSRDFLFSNDYLVTDASVHGGVDESV-------------------------------

DippuMet  683 ----------CSEPGAPSPGSPTHGADFQTSPGNFTLRINQTLLHSPNPNSPLQSMVCIP
TricaMet      ------------------------------------------------------------
DromeMet  689 -------------------------DPHSNTSPPPSLHTRPSVLHRTLTSTLR-------
LocmiMet  749 VITGRCCAGSERMRCSRLSIMSVTGEEKRITSVHGEVVLKRTMSADEENPSCTKKLNVGP
PyrapMet  522 --------------------------DESIMRLSEDVATS-DIWDASVSD--------LD
RhoprMet  534 --------------------------EIRSLHVELEVPIDPRLWESEVEEKVIRGQIQLE

DippuMet  733 QNIMAGMPSPGPQSPARSNSSILDFSPAQLPLTHSPSSAQMASFTVGVGHDSPLDTFAPA
TricaMet      ------------------------------------------------------------
DromeMet      ------------------------------------------------------------
LocmiMet  809 RHCRQRHRIEHPPTEALSVEVNTLPPDELQHIILSRDSSQPT---VLVSQHRPFRTSQPA
PyrapMet  547 DGVLQR-----------------------------HLQLQDK---LDSQEARS-------
RhoprMet  568 NSIQRQERQIFAIENDLTSVPITNTESHIYRSEFTHLRAEHK---KQQQMLKTLQQDREQ
```

```
DippuMet   793 SSHSPLQYTSLSAFGEATSILSPGDSYSSSLGTLDLADLFTGNTNMLLSSSGLYDDIIPE
TricaMet       ------------------------------------------------------------
DromeMet       ------------------------------------------------------------
LocmiMet   866 LQTVTQNVPAVSLSPPQLADITPPPFVMEEPGGMPLVNLSD----TTLSVGCQDPLISPD
PyrapMet       ------------------------------------------------------------
RhoprMet   625 LNVSDI------------------------------------------------------

DippuMet   853 QPTESEQLPLVTMDQRIVQTHRELGTSLQMQRRDINIIEENMQQFPLNIASEMLRPQISQ
TricaMet       ------------------------------------------------------------
DromeMet       ------------------------------------------------------------
LocmiMet   922 HGDSDSFSSLGLPDPENNPEVLDLLEAPELKLNFDTLQEARSSNDPEAPTGPETAATLER
PyrapMet       ------------------------------------------------------------
RhoprMet       ------------------------------------------------------------

DippuMet   913 IKAQQKEHEQELMTLRQDHHNIHCNSRTVCRMNQDVGV----------------------
TricaMet       ------------------------------------------------------------
DromeMet       ------------------------------------------------------------
LocmiMet   982 ILARKHLQLESCMQLQETQMHCIEHDLTQLPLADHVFRSNLTQLQASGPIHARKEERQAS
PyrapMet       ------------------------------------------------------------
RhoprMet       ------------------------------------------------------------

DippuMet   970 ----------------------
TricaMet       ----------------------
DromeMet       ----------------------
LocmiMet  1042 KLHIPVLTPIFMRNNIRSTRYC
PyrapMet       ----------------------
RhoprMet       ----------------------
```

```
DippuKr-h1     1 ------------------------------------------------------------
TricaKr-h1     1 ---------------------------------------------------MPEMVGYYT
DromeKr-h1     1 MTESKNDTKSWAPKQIWIKDVLKKSGTELLDISKSPAKAVAVKKSPAKDSATTKMVYYSA
PyrapKr-h1     1 ------------------------------------------FSANSLWSRVSEMVGYFN
RhoprKr-h1     1 -----------------------MENNKDWIRNFWSSCNKEKQDGNDLWSRVSEMVGYLN
BlageKr-h1     1 --------------------------------------MVGYFNTEDGSGRQHERGGVEC

DippuKr-h1     1 ------------------------------------------------------------
TricaKr-h1    10 ED----------------------PLAIAP------------------------------
DromeKr-h1    61 NQLLIKTEQSSQAQFCLQVPPPLTATTTSVGLGVPPSGGQQEHFELLQTPQQRQMQLQLQ
PyrapKr-h1    19 Q---------EEGRTEP-LPVGADCTLSPL------------------------------
RhoprKr-h1    38 TN-STTSGSGEQAREQPVVAVGADCTLSPL------------------------------
BlageKr-h1    23 TGGPLLPEQHPEEREAPVKRLVCSPDLPVF------------------------------

DippuKr-h1     1 ------------------------------------------------------------
TricaKr-h1    18 --------------------------------VPTVDEARLSVKKVVCSPDLPMPEFPSG
DromeKr-h1   121 DQHQQEQQQFVSYQLAIQQHQKQQQQQQHESITNAAPTAAPSAQRIKTEPVGGFPASAAV
PyrapKr-h1    39 --------------------------------MPQGEPTSEEAKSVVCSPDLPVVFQPPP
RhoprKr-h1    67 --------------------------------GERRE-EEASVKRVVCSPDLS-VFQPP-
BlageKr-h1    53 --------------------------------SLTQAFEEAAAAAVVESTSTTTPTSAPA

DippuKr-h1     1 ------------------------------------------------------------
TricaKr-h1    46 AAPHPDQTIN----IQCQICNKMFATKSAFQAHQRTHTR---------------------
DromeKr-h1   181 VSQVRKPSAS-KPQFKCDQCGMTFGSKSAHTSHTKSHSKNQDLSLNGASGAGVAAPVSTA
PyrapKr-h1    67 VEEAPPDSST-TEVFTCAFCQKSFTQKNAYQNHVRGHN----------------------
RhoprKr-h1    92 -AQPTPADST-NDTLQCTPCNKTFMSRSAHQVHMKTHK----------------------
BlageKr-h1    81 APVAAVTTATGDKVYECSFCHKTFPQKNTYQNHLRSHGK---------------------
                                 Zn1

DippuKr-h1     1 ------------------------------------CGKTFAVPARLTRHYRTHTGEKPY
TricaKr-h1    81 --------------------------ETEDPYRCNICSKTFAVPARLTRHYRTHTGEKPF
DromeKr-h1   240 AIELNDAGLPVGIPKSPTIKPLANVAAGADPYQCNVCQKTFAVPARLIRHYRTHTGERPF
PyrapKr-h1   104 --------------------------KEEDPYQCNFCGKTFAVPARLTRHYRTHTGEKPY
RhoprKr-h1    28 --------------------------EGEDPYQCNCCGKTFAVPARLTRHYRTHTGEKPY
BlageKr-h1   120 --------------------------EGEDPYQCNICGKTFAVPARLTRHYRTHTGEKPY
                                               Zn2

DippuKr-h1    25 QCEYCSKSFSVKENLSVHRRIHTKERPYKCDICARAFEHSGKLHRHMRIHTGERPHKCGV
TricaKr-h1   115 RCEFCNKRFSVKENLSVHRRIHTKERPYKCDVCSRAFEHSGKLHRHMRIHTGERPHKCDV
DromeKr-h1   300 ECEFCHKLFSVKENLQVHRRIHTKERPYKCDVCGRAFEHSGKLHRHMRIHTGERPHKCSV
PyrapKr-h1   138 QCDICKKSFSVKENLSVHKRIHTKERPYKCDICERAFEHSGKLHRHMRIHTGERPHKCGV
RhoprKr-h1   162 QCEYCHKSFSVKENLSVHRRIHTKERPYKCEICERAFEHSGKLHRHMRIHTGERPHKCAV
BlageKr-h1   154 QCEYCSKSFSVKENLSVHRRIHTKERPYKCDICARAFEHSGKLHRHMRIHTGERPHKCGV
                          Zn3                          Zn4

DippuKr-h1    85 CAKTFIQSGQLVIHMRTHTGEKPYVCAA--CGKGFTCSKQLKVHTRTHTGEKPYSCDICG
TricaKr-h1   175 CSKTFIQSGQLVIHKRTHTGEKPYVCTV--CSKGFTCSKQLKVHSRTHTGEKPYSCEICG
DromeKr-h1   360 CEKTFIQSGQLVIHMRTHTGEKPYKCPEPGCGKGFTCSKQLKVHSRTHTGEKPYHCDICF
PyrapKr-h1   198 CNKTFIQSGQLVIHMRTHTGEKPYVCKD--CGKGFTCSKQLKVHNRTHTGERPYSCEICG
RhoprKr-h1   222 CSKTFIQSGQLVIHMRTHTGEKPYVCKD--CGKGFTCSKQLKVHNRTHTGEKPYSCDICG
BlageKr-h1   214 CAKTFIQSGQLVIHMRTHTGEKPYVCAA--CGKGFTCSKQLKVHTRTHTGEKPYSCDICG
                        Zn5                          Zn6

DippuKr-h1   143 KAFGYNHVLKLHQVAHYGEKVYKCTICSQTFTSKKTMEVHIKSHSDPSGAARSPQPPMVS
TricaKr-h1   233 KSFGYNHVLKLHQVAHYGEKVYKCTICNDTFTSKKSMEAHIKSHSEN-------------
DromeKr-h1   420 RDFGYNHVLKLHRVQHYGSKCYKCTICDETFKNKKEMEAHIKGHANEVPDDEAEAAAASA
PyrapKr-h1   256 KSFGYNHVLKLHQVAHYGEKVYKCTICNETFNSKKTMESHIKSHSDN-------------
RhoprKr-h1   280 KSFGYNHVLKLHQVAHYGEKVYKCTICNETFTSKKTMESHIKSHSES-------------
BlageKr-h1   272 KAFGYNHVLKLHQVAHYGEKVYKCTICSQTFTSKKTMEVHIKSHSEP---ARSPQPPLVQ

                       Zn7                         Zn8
```

```
DippuKr-h1  203 STARQQNSDLGESSCASSSSDKENKGDSDCEPPPRIPSLTNEFDPSEVRAISPSSAVPFS
TricaKr-h1  280 APTPPASSTSNESSCSSSTSDKENKDS---------------------------------
DromeKr-h1  480 AASTSAGSSAGSPSLQGVSSNSESSNHSPPSSPPATKKPRQARQPRVSKTVAATLSIPTS
PyrapKr-h1  303 TNREESSGTEMDTSCASSASDKENKES---------------------------------
RhoprKr-h1  327 GRTEEGSTNETETSCASSTSDKENNKSSAEKNGSSGPGGTNTS-----------------
BlageKr-h1  329 SAARQQNGDLGESSCASSSSDKENKGDSDCEPPPRVPSLNSEFDPSEVRSLSPSPAMPFS

DippuKr-h1  263 PPPPHHQQEQE---------------------HHHHHQIQQQQHLQQQQQHQQLQQHHQS
TricaKr-h1  307 ------------------------------------------------------------
DromeKr-h1  540 SPLSPSSLSSTYSPSASSMASPPPTSAHYLPVQMEADALSRDSGVSSAQPAHSTYADEEP
PyrapKr-h1  330 ------------------------------------------------------------
RhoprKr-h1  370 ------------------------------------------------------------
BlageKr-h1  389 SPALHHPPEQEPR-------------------HPHHHHQHQNHHEQMQLQQHHHHLQHQT

DippuKr-h1  302 LPPPQTQNHPNIRELCYSIYCSGNSPYQQR-SSGTSGVNPVLLAVAAAASEGRSLSPLPG
TricaKr-h1  307 -LPMPQDPLSYDSDIRYLIYPRDSG-------YMVPQTGVELLAAAATAT----------
DromeKr-h1  600 TDLSMQQVQGQLPESTVDYYQAPPSLLELQPQPAGLTINPALLEAASIAR----------
PyrapKr-h1  330 --PKPSAEYPNIRELCGYLYRGEKP-----------GGLDPALLAAVAVAT----------
RhoprKr-h1  370 --SSASGNYSNMRELCGYLYRNERA-----------GGLNPALLAAVAVAT----------
BlageKr-h1  430 LP-QQTQPHPNIRELCYSIYCSGNPPYQQREATSSSGVNPVLLAVAAAASEGRSLSPLPG

DippuKr-h1  361 SEDPDDEDMIFRRRQDELPVEPALPVTEPSVYLCPEPLASMQRRS-PYHFQLPPPEYLDE
TricaKr-h1  349 --EREDVVFNIITKNPVMIRQPY--FQTPSVQYTPLNAGDAIRKKVEAVLGVG-------
DromeKr-h1  650 --RHDDNDDQVQDEDVHAAAWQMMQLCRGHGSLPPTEQPAPSHQPQVPTLHVSDLAANYD
PyrapKr-h1  368 SENSAE-----YRPSPEHVLETP----DPPVYLVPT------------------------
RhoprKr-h1  408 SENGPE-----YRPSPDHVLETPSGLAEPPVYLVPEPVACIQRKR-SYTPELILP-----
BlageKr-h1  489 SEDHDDEDMVFRRRQEEMS-EPALPVTEPSVYLCPEPLASMQRRASPYHFQLPPPDYPEE

DippuKr-h1  420 PVHINLPLTPAYSSSSSSSSS--SGSRRPRPEEPEEHEIPEDH-DPLLTPPSSNPVSPVP
TricaKr-h1  398 ---------------------------EMPSSEEEN----------ILTPPSSNPVSPAA
DromeKr-h1  708 DTHEATVLIEHFKRGDLARHGLHKGYAPVPKYESALPNPDVVRRVEAAIGLRSSTESPER
PyrapKr-h1  395 ---------------------------SIRPEEVE-----------------------VE
RhoprKr-h1  457 --------------------------DMMTPEEEEE----------LLTPPSSNPVSPVP
BlageKr-h1  548 PMQISLPLTPAYSTSSSSSSSSSVGGRRRRPLESEEHEMEQ---DPLLTPPSSNPVSPVP

DippuKr-h1  477 SSSPDPIDLATVSREALILPPRKRSKMILKSRK---------------------------
TricaKr-h1  421 SPVSMSSP-----DRELSLPPRKRSRMILKSLEETID-----------------------
DromeKr-h1  768 SSSPESDSLMMADRNVMTLPLRKRKHYMNKGDDGQVDSEKASGDGTSAAGGAASVGAGDG
PyrapKr-h1  405 DSS----------RELISLPPRKRSKAILKSMEREE------------------------
RhoprKr-h1  481 SSSPEPVDL----KEVISLPPRKRTKAILKSMESETSS----------------------
BlageKr-h1  605 SSSPDPIDLATVSREALILPPRKRSKMILKSMETAEPE----------------------
                                  LP(L/P)RKR

DippuKr-h1      ------------------
TricaKr-h1  453 --LSPVRYSSVIQYAGAS
DromeKr-h1  828 PGSKVMRMSSVIQFAKAS
PyrapKr-h1  431 ---APVRYSSVIHYAKAS
RhoprKr-h1  515 --TPPVRYSSVIHYAKAS
BlageKr-h1  643 --VVSVRSRSVIHYARAS
```

```
DippuBr-C    1 MADTQHFCLRWNNYQSSITSAFENLRDDEDFVDVTLACDGKSLKAHRVVLSACSPYFREL
TricaBr-C    1 MVDTQHFCLRWNNYQSSITSAFENLRDDEDFVDVTLACDGKSLKAHRVVLSACSPYFREL
DromeBr-C    1 MDDTQHFCLRWNNYQSSITSAFENLRDDEAFVDVTLACEGRSIKAHRVVLSACSPYFREL
PyrapBr-C    1 ------------------------------FVDVTLACDGKSLKAHRVVLSACSPYFREL
BlageBr-C    1 MADTQHFCLRWNNYQSSITSAFENLRDDEDFVDVTLACDGKSLKAHRVVLSACSPYFREL

DippuBr-C   61 LKSTPCKHPVIVLQDVAFADLHALVEFIYHGEVNVHQRNLSSFLKTAEVLRVSGLTQQAE
TricaBr-C   61 LKSTPCKHPVIVLQDVAWTDLHALVEFIYHGEVNVHQRSLSSFLKTAEVLRVSGLTQQHG
DromeBr-C   61 LKSTPCKHPVILLQDVNFMDLHALVEFIYHGEVNVHQKSLQSFLKTAEVLRVSGLTQQQA
PyrapBr-C   31 LKSTPCKHPVIVLQDVMFEDLHALVEFIYHGEVNVRQRSLSSFLKTAEVLRVSGLTQQSG
BlageBr-C   61 LKSTPCKHPVIVLQDVAFADLHALVEFIYHGEVNVHQRNLSSFLKTAEVLRVSGLTQQAE

                   BTB
DippuBr-C  121 DRDE-FPAQLQ-----------------GSRTHQQSFSDELVEDALFTS-PPSPPPPSQH
TricaBr-C  121 DDRE-QLAQVQSLVRS--------QQSTPTSNHHPSFTEKLVEDALFTS-PSSPP-----
DromeBr-C  121 EDTHSHLAQIQNLANSGGRTPLNTHTQSLPHPHHGSLHDDGGSSTLFSRQGAGSPPP--T
PyrapBr-C   91 -----------------------------------AVSMGLDKGAGLAD-GRGSP-----
BlageBr-C  121 DRDE-FPAQLQ-----------------GSRCHQQSFSDKLVEDALFTS-PPSPPPPSQH

DippuBr-C  162 SNATSGGAT---------------------------------------------------
TricaBr-C  166 -----HGATVNQLLRRAALQH------RRERRISSDPEGD----------HKRPRPDHII
DromeBr-C  179 AVPSLPSHINNQLLKRMAMMH-------RSSAAAAAEETSHAFKRLRGSDNSLPLSGAVG
PyrapBr-C  110 -----------HHPRKPQHKH-------RDRPVSTPESTE--------------------
BlageBr-C  162 SNATSGGATVNQLLRRAAAAAIASSSGRRERHNSAEQQSDSSEQSQHSAPSKRARTSANV

DippuBr-C      ------------------------------------------------------------
TricaBr-C  205 GNNNNNELNLS----HTQPPQTQPADFS----------PSAMKNNALNLNMNSSSTTTTQ
DromeBr-C  232 SGSNNNSPDLPPLHARSASPQQTPADFSTIKHHNNNNTPPLKEEKRNGPTGNGNSGNGNG
PyrapBr-C  132 -NQDINPMKRP----KNEPPN-SYSERS----------PPMVDMEEPP--MDFSTTSGNN
BlageBr-C  222 ENSNHNNGETT----SASTNQVSATDFS----------SSPFSSKQA----NQGSTTANN

DippuBr-C      ------------------------------------------------------------
TricaBr-C  251 NEANNGITS-TERENSPCSPSPTLNSRLNEENVKSEPMELLCST----NQEENSNDSGDV
DromeBr-C  292 NGNGASNGNGISISDKLGSLTPSPLARAGADDVKSEPMDMVCSNNNANANDEHSNDSTGE
PyrapBr-C  174 NNNNNNNNN-NSREKSDGSPTRSD--------IKPEPIELTPHIG---NEKEDSGDSGGE
BlageBr-C  264 NRKADSEGN-AASGDGDSSPSPTS-GMLDG--VKSEPLELLCGT----AEMDNSNDSVEA

DippuBr-C      ------------------------------------------------------------
TricaBr-C  306 ANDNGPNNLPQGPHSGSSAGDH--DEHDS----PIGPYLTPSESKLFATA-AGSFNFSMA
DromeBr-C  352 HDANRSSSGDGGKGSLSSGNDEEIGDGLASHHAAPQFIMSPAENKMFHAA---AFNFPN-
PyrapBr-C  222 LEPGPP-------HLPLQLPDH--DDSMQ----SAHSYLD---SKLFAAG--AGFQFSMA
BlageBr-C  316 TGGDIPSAGHQ--QSSLSAGDH--DDHDSIQGHPASAYLA-SESKLFASATSGSFNFSMA

DippuBr-C      ------------------------------------------------------------
TricaBr-C  359 ALAADPTGLGGLN-QSLQAN-ADSLAGTSQESSGGGRPGRTAFCDICNKTFSRFWSLQRH
DromeBr-C  408 ---IDPSALLGLNTQLQQSG---DLAVSPQG----NSPKKLFSCQLCGKLLCSKASLKRH
PyrapBr-C  264 AALAADS-LAGLNSQGHGLGNSDGLAGTSQQ-----GLRKMFTCVACKKVLCSKASLKRH
BlageBr-C  371 ALAADHPALPGIGGQGLQGS-TDGSAGTSSQ----GSPKKVFTCQLCGKVLCSKASLKRH

DippuBr-C      ---------------------------------------------------------
TricaBr-C  417 LSDTHFYTPQNLQCNVCGRSYRSRNSLVSHRSQYHREGN-EIKFENDCFI-------
DromeBr-C  458 IADKHAVRQEEYRCAICERVYCSRNSLMTHIYTYHKSRPGEMEMKDIKLYNQFNSSI
PyrapBr-C  318 VADKHASRHEEYRCAI-----------------------------------------
BlageBr-C  426 VADKHAERQEEYRCIICERVYCSRNSLMTHIYTYHKTRTGEVDIK---FF-------

                  Zn Z2
```
