# Supplementary figures and images for: Methoprene-Tolerant (Met) Knockdown in the Adult Female Cockroach, Diploptera punctata Completely Inhibits Ovarian Development
Source: PLoS One. 2014 Sep 8;9(9):e106737. doi: 10.1371/journal.pone.0106737 (PMC4157775; doi:10.1371/journal.pone.0106737)

Control  
*HMGR-JHAMT* RNAi

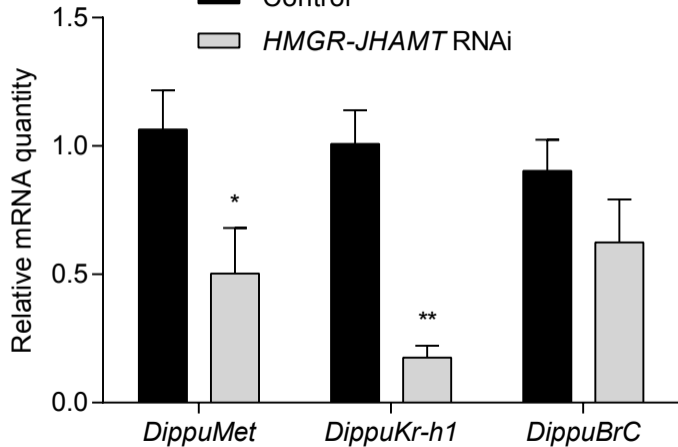

Supplement: Figure S2 — Knockdown of DippuHMGR-DippuJHAMT by RNAi results in significant downregulation of DippuMet , DippuKr-h1 , DippuVg. Measurements were taken from the fat body on day 4 of the first gonadotropic cycle. Data points represent the mean 5 individual animals, (n = 5) and three technical replicates, normalized to Tub and EF1α. Vertical error bars indicate SEM. (PDF) [file pone.0106737.s002.pdf]

|            |   |   |   |   |
|------------|---|---|---|---|
| Acetone    | - | + | + | - |
| Methoprene | - | - | - | + |

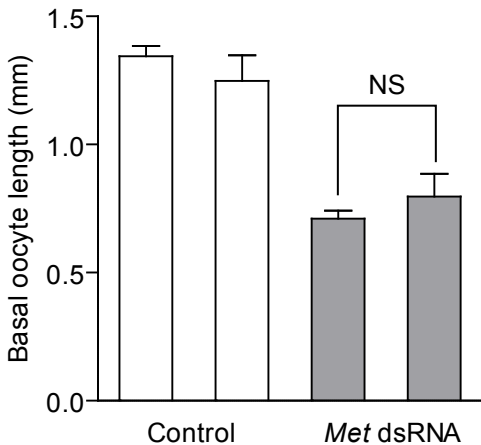

Supplement: Figure S3 — Effect of topical application of methoprene on oocyte growth in DippuMet dsRNA treated animals versus control animals (n = 5). Plus sign (+) indicates whether animals were treated with acetone or 100 µg of methoprene. (PDF) [file pone.0106737.s003.pdf]
