# Supplementary material for: Methoprene-Tolerant (Met) Knockdown in the Adult Female Cockroach, Diploptera punctata Completely Inhibits Ovarian Development
Source: PLoS One. 2014 Sep 8;9(9):e106737. doi: 10.1371/journal.pone.0106737 (PMC4157775; doi:10.1371/journal.pone.0106737)
Supplement: Table S1 — Degenerate primer and RACE primer sequences for cloning partial DippuMet , DippuKr-h1 and DippuBr-C cDNAs. (DOCX) [file pone.0106737.s004.docx]

**Supporting Table S1** Degenerate primer and RACE primer sequences for cloning partial *DippuMet*, *DippuKr-h1* and *DippuBr-C* cDNAs.

| **Name** | **Symbol** | **Degenerate/RACE primer sequences** | **Accession number (GenBank)** |
| --- | --- | --- | --- |
| Methoprene tolerant | *DippuMet* | F 5'- AARCARMGNMGNSAIAARYTIAA -3'  R 5'- ACIARIGTRTTIAYRCANAYRAA -3'  Fn 5’- CACTCCCCCAGGAAGNTNGAYAARAC-3’  Rn 5’- ACIARIGTRTTIAYRCANAYRAA-3’  5’Race 5’- CTGCGTAATCTCACTGCGAGGC-3’  3’Race 5’-CCGTGGAAAGGACAGCTACG-3’ | KJ564130 |
| Krüppel  homolog 1 | *DippuKr-h1* | F 5'- GARGATCCMTACCRKTGYAAYATYTG -3'  R 5'- CGTTTCCAWRGAYTTBARWATCAT -3' | KJ564131 |
| Broad complex | *DippuBr-C* | F 5'- ATGGCAGAYACRCARCATTTTTGC -3'  R 5'- TCGATGACGTCCCTGCTGAGCCAT -3' | KJ564132 |
