# Supplementary material for: Methoprene-Tolerant (Met) Knockdown in the Adult Female Cockroach, Diploptera punctata Completely Inhibits Ovarian Development
Source: PLoS One. 2014 Sep 8;9(9):e106737. doi: 10.1371/journal.pone.0106737 (PMC4157775; doi:10.1371/journal.pone.0106737)
Supplement: Table S2 — Oligonucleotide sequences for primers used in q-RT-PCR for reference and target genes. Efficiencies and R2 values are indicated. (DOCX) [file pone.0106737.s005.docx]

**Supporting Table S2** Oligonucleotide sequences for primers used in q-RT-PCR for reference and target genes. Efficiencies and R² values are indicated.

| **Reference genes** | **F-primer** | **R-primer** | **amplicon size (bp)** | **Efficiency (%)** | **R^2^** |
| --- | --- | --- | --- | --- | --- |
| ***DippuTubulin*** | 5'-AAATTACCAACGCTTGCTTTGAA-3' | 5'-TGGCGAGGATCGCATTTT-3' | 58 | 95.1 | 0.993 |
| ***DippuEF1-α*** | 5'-TCGTCTTCCTCTGCAGGATGTCT-3' | 5'-GGGTGCAAATGTCACAACCATACC-3' | 109 | 99.2 | 0.994 |
| **Target genes** | **F-primer** | **R-primer** | **amplicon size (bp)** | **Efficiency (%)** | **R^2^** |
| ***DippuMet*** | 5’-GACAAGATGAACGCCCATATCA-3’ | 5’-GTCTAAGGAAGGCAGCAGTAAG-3’ | 115 | 97.6 | 0.992 |
| ***DippuKr-h1*** | 5’-ACACAGCGGCAAGTTACA-3’ | 5’-AAGTTGACCGCTCTGGATAAA-3’ | 100 | 99.8 | 0.990 |
| ***DippuBr-C*** | 5’-GCAAGCAGAGGATAGAGATGAG-3’ | 5’-CGGTGATGGAGGTGATGTAAA-3’ | 115 | 100 | 0.991 |
| ***DippuVg*** | 5’-AAAGGTGTCCTCAGCCAGC-3’ | 5’-TCCTCCATCTCGGATTGGGA-3’ | 105 | 95.1 | 0.998 |
