# Supplementary material for: Methoprene-Tolerant (Met) Knockdown in the Adult Female Cockroach, Diploptera punctata Completely Inhibits Ovarian Development
Source: PLoS One. 2014 Sep 8;9(9):e106737. doi: 10.1371/journal.pone.0106737 (PMC4157775; doi:10.1371/journal.pone.0106737)
Supplement: Table S3 — Nucleotide sequences of primers used in making the dsRNA constructs. (DOCX) [file pone.0106737.s006.docx]

**Supporting Table S3** Nucleotide sequences of primers used in making the dsRNA constructs.

| **RNAi constructs** | **F-primer** | **R-primer** |
| --- | --- | --- |
| *DippuMet1* | 5'-**TAATACGACTCACTATAGGGAG** AACATCTTGTTCGTGTCCCATAC -3' | 5'-**TAATACGACTCACTATAGGGAGA** CTGTTCTCTGACTCAACCGAAG -3' |
| *DippuMet2* | 5'-**TAATACGACTCACTATAGGGAGA** ACTCGGCACCTTATTGATGGCC -3’ | 5'- **TAATACGACTCACTATAGGGAGA** CATCTTCCGGCACGAGGGT-3’ |
| -pJET (control) | 5'- **TAATACGACTCACTATAGGGAGA** TTGCGCTCACTGCCAATTGC-3’ | 5'- **TAATACGACTCACTATAGGGAGA** CTGGCCTTTTGCTCACATGTT-3’ |
